# Inflammatory mediator ultra-low-molecular-weight hyaluronan triggers necrosis of B-precursor leukemia cells with high surface CD44 expression

**DOI:** 10.1038/cddis.2017.249

**Published:** 2017-06-01

**Authors:** Shin Kasai, Yoshiyuki Furuichi, Norie Ando, Keiko Kagami, Masako Abe, Takaya Nakane, Kumiko Goi, Takeshi Inukai, Sei Saitoh, Shinichi Ohno, Shogo Okazaki, Osamu Nagano, Hideyuki Saya, Kanji Sugita

**Affiliations:** 1 Departments of Pediatrics, Graduate School of Medicine, University of Yamanashi, Yamanashi, Japan; 2 Departments of Anatomy and Molecular Histology, Graduate School of Medicine, University of Yamanashi, Yamanashi, Japan; 3 Division of Gene Regulation, Institute for Advanced Medical Research, School of Medicine, Keio University, Tokyo, Japan

## Abstract

Acute lymphoblastic leukemia (ALL) with *mixed lineage leukemia* (*MLL*) gene rearrangements (*MLL*+ALL) has a dismal prognosis and is characterized by high surface CD44 expression. Known that CD44 has the specific binding sites for a natural ligand hyaluronan (HA), we investigated biological effects of HA with different molecular sizes on *MLL*+ALL cell lines, and found that the addition of ultra-low-molecular-weight (ULMW)-HA strongly suppressed their thymidine uptakes. The *MLL*+ALL cell line lacking surface CD44 expression established by genome editing showed no suppression of thymidine uptake. Surface CD44-high B-precursor ALL cell lines other than *MLL+*, but not T-ALL cell lines, were also suppressed in their thymidine uptakes. The inhibition of thymidine uptakes was because of induction of cell death, but dead cells lacked features of apoptosis on cytospin smears and flow cytometric analysis. The cell death was neither blocked by pan-caspase inhibitor nor autophagy inhibitor, but was completely blocked by necrosis inhibitor necrostatin-1. Necrotic cell death was further supported by a marked release of a high-mobility protein group B1 and morphological changes on transmission electron microscopy. Elevation of intracellular reactive oxygen species production suggested a role for inducing this necrotic cell death. ULMW-HA-triggered cell death was similarly demonstrated in surface CD44-high primary B-precursor leukemia cells. Assuming that ULMW-HA is abundantly secreted at the site of infection and inflammation, this study sheds light on understanding the mechanism of a transient inflammation-associated remission of leukemia. Further, the CD44-targeting may become an effective approach in future for the treatment of refractory B-precursor ALL by its capability of predominantly eradicating CD44-high leukemia-initiating cells.

*Mixed lineage leukemia* (*MLL*) gene rearrangements are frequently observed in infantile acute lymphoblastic leukemia (ALL) and therapy-related second leukemia.^1, 2^ ALL with *MLL* gene rearrangements (*MLL*+ALL) has a unique gene profile clearly distinguishable from those of other types of ALL and acute myeloid leukemia (AML), and that the *Fms-like tyrosine kinase 3* (*FLT3*) and *CD44* genes are the most differentially expressed at very high levels.^3^ We previously reported that triggering of *MLL*+ALL cells by FLT3 ligand, abundantly secreted from bone marrow stromal cells, induces their cell cycle arrest showing resistance to chemotherapy, which should lead to the persistent formation of minimal residual disease accountable for the refractory nature of the disease.^4^ It should be now important to characterize the role of CD44 in *MLL*+ALL to possibly find a new therapeutic strategy for overcoming this dismal disease.

CD44 is a widely distributed type I transmembrane glycoprotein expressed on hematopoietic cells, various types of epithelial cells, fibroblasts and endothelial cells^5^ and mediates various physiological and pathological cellular functions such as extracellular binding, cell migration, hematopoiesis, lymphocyte homing as an adhesion molecule in cell–cell and cell–substrate interactions.^6^ It is also expressed on a wide range of hematological and non-hematological tumors and is recently attracting attention as a cancer stem cell (or a cancer-initiating cell) marker for several types of malignancies^7, 8, 9, 10^ and one of key molecules involving epithelial–mesenchymal transition.^11^ CD44 has the specific binding sites for hyaluronan (HA).^9^ HA, a glycosaminoglycan, is a ubiquitous component of the extracellular matrix (ECM) and has a fundamental role in maintaining the ECM architecture. High-molecular-weight (HMW)-HA (10^3^–10^4^ kD), which exists as ECM in tissues, is incorporated into cells in a CD44-dependent manner, and then is secreted from them as low-molecular-weight (LMW)-HA (<500 kD) to ultra-LMW (ULMW)-HA (<10 kD) under normal and pathological conditions,^12^ particularly at the site of infection and inflammation.^13, 14^

The purpose of this study is to investigate the biological events elicited after ligand stimulation of CD44 by using HA with distinct molecular sizes in B-precursor ALL including *MLL*+ALL, and to determine whether or not these events are also observed in other ALL cell lines and primary leukemia cells with high surface CD44 expression.

## Results

### Surface CD44 expression and changes in thymidine uptake after ULMW-HA stimulation in *MLL*+ALL cells

**Figure 1:**
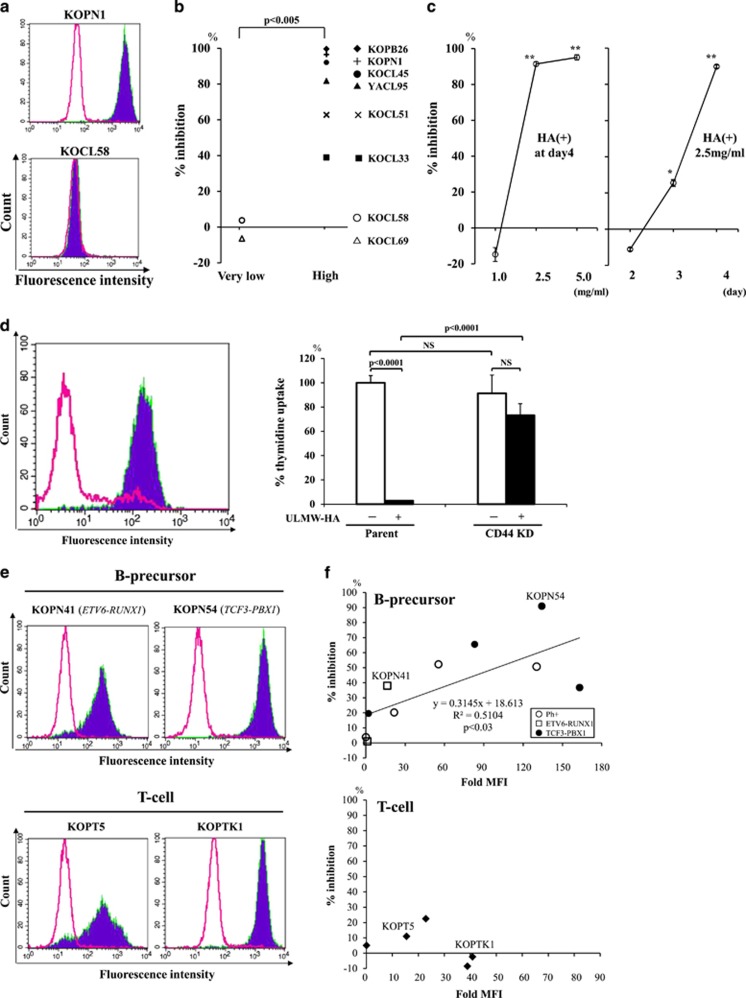
Surface CD44 expression and effects of ULMW-HA on *MLL*+ALL cell lines. (**a**) Surface CD44 expression. *MLL*+ALL cell lines were stained with normal IgG (open cytograms) or anti-CD44 mAb (filled cytograms). Cytograms of CD44-high KOPN1 and CD44-very low KOCL58 cell lines were representatively shown. (**b**) % Inhibition of thymidine uptake. Cell lines were divided into CD44-high (>95% positive; *n*=6) and CD44-very low (<5% positive; *n*=2) groups, and compared % inhibition of thymidine uptake at day 4 after ULMW-HA stimulation (2.5 mg/ml). The CD44-high group cell lines showed significantly higher inhibition (*P*<0.005 by Student’s *t*-test). (**c**) Changes in % inhibition of thymidine uptake. The *MLL*+ALL cell line expressing CD44 at high levels, KOPB26, was cultured (0.5 × 10^5^ per well) in triplicate in the presence or absence of various concentrations (1.0, 2.5 and 5.0 mg/ml) of ULMW-HA for 4 days (left panel), and at a concentration of 2.5 mg/ml of ULMW-HA for 2, 3 and 4 days (right panel), and 5 h-thymidine uptake was examined. The % inhibition in the presence of HA was calculated as {1-[(cpm of HA+)/(cpm of HA-)]} × 100. The results are representative from three separate experiments and are shown as mean±S.E. The significance of differences between HA- and HA+ culture conditions at day 4 (left panel) and at days 2, 3 and 4 (right panel) was analyzed by Student’s *t*-test (*P<0.005, **P<0.001). (**d**) Establishment of the CD44 knockdown (KD) cell line and its thymidine uptake after ULMW-HA stimulation. The established cell line KOPB26^CD44KD^ by genome editing (open cytogram) showed surface CD44 expression at very low levels (<10%) when compared with the parent cell line KOPB26 (filled cytogram) by flow cytometric analysis (left panel). The parent KOPB26 and KOPB26^CD44KD^ cell lines were cultured in triplicate in the presence or absence of ULMW-HA (2.5 mg/ml) for 4 days and thymidine uptake was examined (right panel). The results are representative from three separate experiments and are shown as % thymidine uptake (mean±S.E.) when thymidine uptake of the parent cell line in the absence of HA is regarded as 100%. The differences were analyzed by Student’s *t*-test. NS, not significant. (**e**) Surface CD44 expression in B-precursor and T-cell leukemia cell lines. Leukemia cell lines were stained with normal IgG (open cytograms) or anti-CD44 mAb (filled cytograms). Cytograms of surface CD44 expression in two B-precursor (KOPN41, KOPT54) and two T-cell (KOPT5, KOPTK1) leukemia cell lines were representatively shown. (**f**) Effects of ULMW-HA on thymidine uptakes of B-precursor and T-cell leukemia cell lines. Ten B-precursor ALL cell lines (four Philadelphia chromosome-positive, four TCF3-PBX1 positive and two ETV6-RUNX1) and five T-ALL cell lines were cultured (0.5 × 10^5^ per well) in the presence or absence of ULMW-HA (2.5 mg/ml) for 4 days, and the % inhibition in the presence of ULMW-HA was calculated. The *X* and *Y* axes represent fold MFI of surface CD44 expression and % inhibition of thymidine uptake, respectively

We first examined surface CD44 expression in eight *MLL*+ALL cell lines, and found that six expressed CD44 at high levels; positive staining >95%, fold mean fluorescence intensity (MFI) >24.0, whereas two expressed CD44 at very low levels; positive staining <5%, fold MFI <1.2. Flow cytograms of representative cell lines are shown in Figure 1a. To address HA-mediated biological effects on *MLL*+ALL cells, we next examined thymidine uptake after 4-day culture with or without various concentrations of ULMW-HA or HMW-HA, and found that thymidine uptake in CD44-high cell lines was markedly suppressed after culture with 2.5 mg/ml of ULMW-HA (% inhibition; 78.6±9.6%, mean±S.E.), but substantially not affected (% inhibition; −1.0±5.1%) in CD44-very low cell lines. This difference in % inhibition of thymidine uptakes between two groups was highly significant (*P*<0.005) (Figure 1b). HMW-HA did not show any effect on thymidine uptake in any of the eight cell lines tested (data not shown). As depicted in Figure 1c, the representative CD44-high cell line KOPB26 exhibited marked suppression of thymidine uptake after incubation with 2.5 mg/ml ULMW-HA in a time-dependent manner, reaching a >90% decrease at day 4. Subsequent analyses were thus mainly done at a concentration of 2.5 mg/ml of ULMW-HA using this cell line KOPB26, unless specifically stated.

### High surface CD44 expression is required to ULMW-HA-triggered inhibition of thymidine uptake in *MLL*+ALL cells

It is known that RHAMM, a unique member of hyaladherins with a variable distribution on the cell surface, also functions as a receptor for HA.^15^ To address the possibility that ULMW-HA-triggered inhibition of thymidine uptake may be mediated in part through the interaction with RHAMM, we examined surface expression of RHAMM on several CD44-high cell lines by flow cytometry, and found that they did not express RHAMM at all on their surfaces (data not shown). Next, to conclude that ULMW-HA-triggered inhibition of thymidine uptake is specifically mediated by interaction with surface CD44, we attempted to generate the KOPB26 cell line lacking the surface CD44 expression by genome editing using the CRISPR/Cas9 system.^16^ As shown in Figure 1d (left panel), we successfully established the cell line expressing CD44 at very low levels (CD44KD). Genomic DNA sequence (*CD44 exon 2*) analysis of this cell line showed insertion/deletion of nucleotides in six of seven clones examined and two of which resulted in formation of stop codon (Supplementary Figure 1). Of note, this cell line exhibited substantially no inhibition of thymidine uptake after ULMW-HA stimulation (Figure 1d**,** right panel), demonstrating an essential role of surface CD44 expression for ULMW-HA-triggered inhibition of thymidine uptake.

### ULMW-HA-triggered inhibition of thymidine uptake is observed in other B-precursor ALL cell lines, but not in T-ALL cell lines, with high CD44 expression

To determine whether or not ULMW-HA-triggered inhibition of thymidine uptake is also observed in other ALL cell lines, we performed similar experiments after ULMW-HA stimulation by using 10 B-precursor ALL cell lines (four Philadelphia chromosome-positive, four *TCF3-PBX1* positive, two *ETV6-RUNX1* positive), and five T-ALL cell lines with a variety of surface CD44 expression. Flow cytograms of CD44 expression in representative cell lines were shown in Figure 1e. Of note, inhibition of thymidine uptakes after ULMW-HA stimulation were also observed in B-precursor ALL cell lines with distinct genetic back grounds, and % inhibition significantly correlated to the fold MFI of surface CD44 expression (Figure 1f, upper panel). In contrast, T-ALL cell lines did not show any inhibition of thymidine uptakes irrespective of surface CD44 expression (Figure 1f, lower panel). These results suggest that ULMW-triggered inhibition of thymidine uptake is not an event restricted to *MLL*+ALL but a universally observed biological event in B-precursor ALL with high CD44 expression.

### Analysis of CD44 splice variants

**Figure 2:**
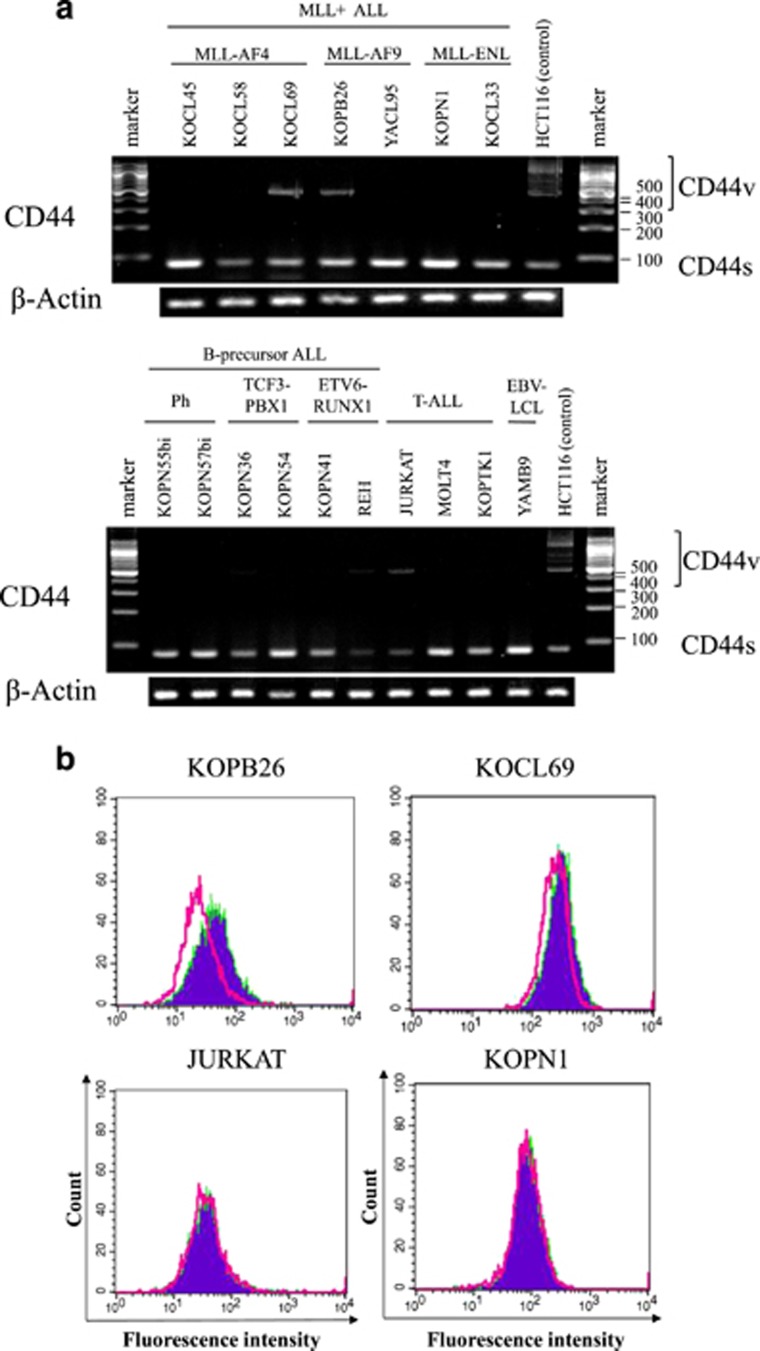
Analysis of CD44 splice variants. (**a**) RT-PCR analysis of CD44 splice variants. mRNAs were extracted from *MLL+* ALL (*n*=7), other B-precursor ALL (*n*=6), EBV-transformed LCL (*n*=1) and T-ALL cell lines (*n*=3), and RT-PCR were performed using primers described in Materials and methods section. The colorectal carcinoma cell line HCT116 was used for positive control. (**b**) Flow cytometric analysis of CD44v9 expression. Three CD44v mRNA-positive cell lines (KOPB26, KOCL69 and JUTKAT) and one CD44v mRNA-negative cell line (KOPN1) were stained with normal rat IgG (open cytograms) or rat anti-human CD44v9 antibody (filled cytograms) and labeled with FITC-conjugated second antibody

Primary transcripts of the CD44 gene can be alternatively spliced to produce a variety of mRNAs. The standard form of CD44 (CD44s) mRNA contains sequences from at least 10 genomic exons, whereas variant mRNAs contain sequences from one to more additional exons (v1–10). Predominant expression of a specific CD44 variant such as CD44v8-10 has been reported in several human carcinomas.^17^ To explore the implication of CD44 splice variants in ULMW-HA-triggered inhibition of thymidine uptake, we performed reverse transcription-PCR analysis of CD44 splice variant RNAs in B-precursor and T-ALL cell lines as described previously.^17^ The colorectal carcinoma cell line HCT116 was used for positive control. As shown in Figure 2a, all of the ALL cell lines examined expressed CD44s most commonly detected in hematologic malignancies. Two (KOPB26, KOCL69) of 17 B-precursor cell lines and 1 (JURKAT) of 3 T-ALL cell line examined expressed CD44v in addition to CD44s. Judging from a molecular size (479 bp) of variants in these three cell lines identical to that in the control cell line HCT116, which is known to express CD44v8-10, all variant mRNAs detected in ALL cell lines were postulated as CD44v8-10. To add evidence to this, we next performed flow cytometric analysis using mAb specifically recognizing CD44v9 isoform. As shown in Figure 2b, the CD44v9 isoform was detected at low levels in CD44v mRNA-positive KOPB26 and KOCL69 cell lines, but not in JURKAT presumably because of a very low translational level. Surface CD44v9 expression was not detected in CD44v mRNA-negative cell lines examined. These results suggest that the presence or absence of CD44v8-10 on the surface is not associated with the magnitude of thymidine uptake inhibition after ULMW-HA stimulation in B-precursor and T-cell ALL cells.

### Cell cycle analysis after ULMW-HA stimulation

**Figure 3:**
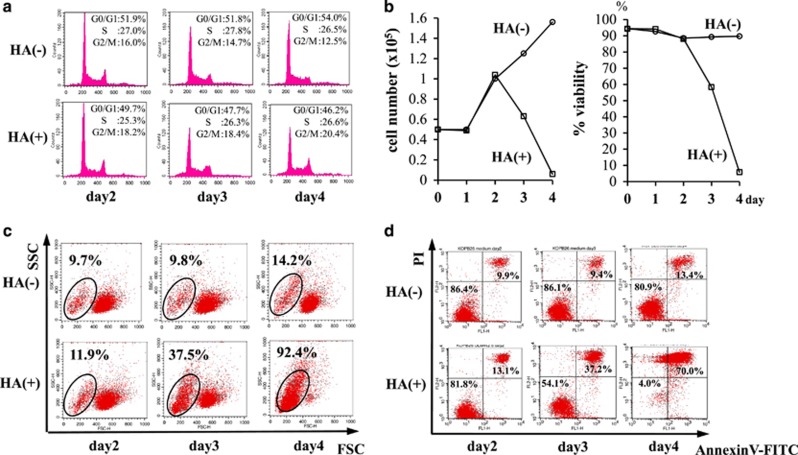
Analysis of cell death after ULMW-HA stimulation. (**a**) Cell cycle analysis. KOPB26 cells (0.5 × 10^5^ per well) were cultured in the presence or absence of ULMW-HA (2.5 mg/ml) for up to 4 days, and cell cycle analysis was performed by the PI staining at days 2, 3 and 4. (**b**) Changes in cell number and viability. KOPB26 cells (0.5 × 10^5^ per well) were cultured in the presence or absence of ULMW-HA (2.5 mg/ml) up to 4 days, and changes in cell number (left panel) and viability (right panel) were examined in duplicate by the dye exclusion test at days 1, 2, 3 and 4. The results are representative from three separate experiments and are shown as the mean. (**c**) Flow cytometric analysis on scatter cytograms. KOPB26 cells (0.5 × 10^5^ per well) were cultured in the presence or absence of ULMW-HA (2.5 mg/ml) for up to 4 days, and cytograms (FSC *versus* SSC) were examined at days 2, 3 and 4. (**d**) Flow cytometric analysis of cell death using annexin V/PI staining. KOPB26 cells (0.5 × 10^5^ per well) were cultured in the presence or absence of ULMW-HA (2.5 mg/ml) for up to 4 days, and flow cytometric analysis of cell death was performed by annexin V/PI staining at days 2, 3 and 4

To examine whether ULMW-HA affects cell cycle progression in CD44-high cell lines, we next performed cell cycle analysis in KOPB26 cells, and found no significant changes in cells in the G0/G1, S and G2/M phases and little increase in the subdiploid apoptotic population after ULMW-HA stimulation (Figure 3a), suggesting that ULMW-HA-mediated inhibition of thymidine uptake is not elicited by cell cycle arrest, but rather induction of cell death other than apoptosis.

### Induction of cell death after ULMW-HA stimulation

To confirm the induction of cell death after ULMW-HA stimulation, we first examined changes in cell number and viability by the dye exclusion test in KOPB26 cells, and found a gradual decrease in cell numbers and viabilities reaching <10% at day 4 (Figure 3b). Of importance, induction of cell death was similarly observed by dye exclusion test when KOPB26 cells were precultured for 8 h in the presence of ULMW-HA and then cultured for 4 days in the absence of ULMW-HA, suggesting that biological effect could be elicited once ALL cells are exposed to a considerable concentration of ULMW-HA.

**Figure 4:**
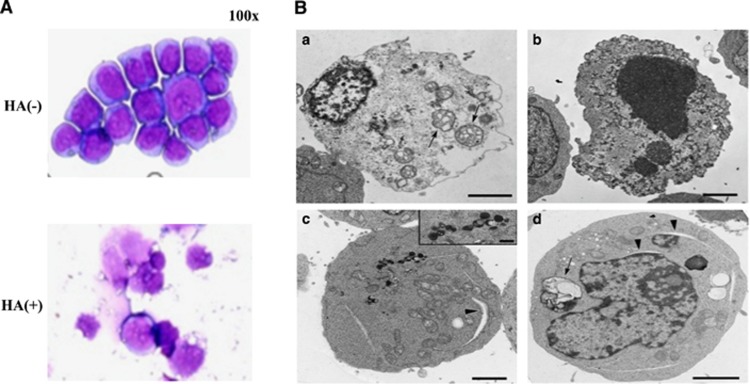
Morphological observation after ULMW-HA stimulation. (**A**) Cytospin smears. KOPB26 cells (0.5 × 10^5^ per well) were cultured in the presence or absence of ULMW-HA (2.5 mg/ml) for 4 days. Cytospin smears were stained with Wright–Giemsa method and observed by light microscopy. (**B**) TEM. KOPB26 cells (0.5 × 10^5^ per well) were cultured in the presence or absence of ULMW-HA (2.5 mg/ml) for 3 days, and then observed by TEM. (a and b) Dying cells lost their plasma membrane integrity and had condensed nuclei lacking nuclear membranes and swollen mitochondria with vacuolar cristae (arrows). (c and d) Living cells showed widely opened ERs (arrowheads), autolysosomes (c, inset), and autophagosomes (arrow). Bars, 2 *μ*m. Bar of inset in (**c**), 500 nm

We also checked the FSC/SSC cytograms on a flow cytometer, and found a gradual increase in the low FSC/wide SSC population (>90% of cells at day 4), which was suspected of being dying cells (Figure 3c). We next performed the annexin V and propidium iodide (PI) stainings on a flow cytometer, and detected a gradual increase in cells doubly stained with annexin V and PI (Figure 3d). At day 4, the percentages of double positive (dying) and negative (living) populations were 70% and 4%, respectively. Cytospin smears at day 4 revealed a large number of shrunken dying cells and a small number of swollen cells with or without vacuoles by light microscopy (Figure 4A). This induction of cell death was not observed in the cell line lacking the surface CD44 expression by genome editing (data not shown).

### Nature of cell death after ULMW-HA stimulation

**Figure 5:**
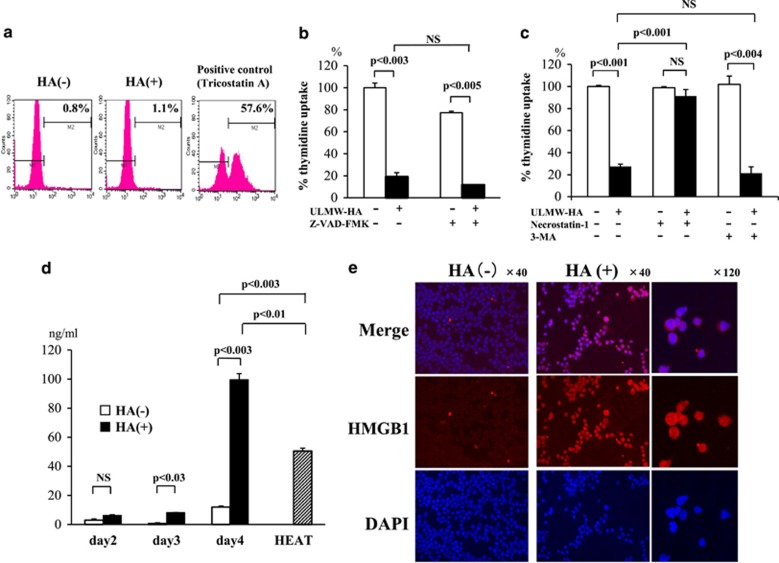
Analysis of nature of cell death after ULMW-HA stimulation. (**a**) Expression of cleaved caspase-3. KOPB26 cells (0.5 × 10^5^ per well) were cultured in the presence or absence of ULMW-HA (2.5 mg/ml) for 4 days, stained with anti-cleaved caspase-3 antibody after permeabilization, and analyzed by flow cytometry. Cells treated with the histone deacetylase inhibitor Tricostatin A (30 ng/ml) were used as a positive control for caspase-3 activation. (**b**) Effect of pan-caspase inhibitor on thymidine uptake. KOPB26 cells (0.5 × 10^5^ per well) were cultured in triplicate in the presence or absence of ULMW-HA (2.5 mg/ml) with or without Z-VAD-FMK (100 *μ*M) for 4 days, and thymidine uptake was examined. The results are representative from three separate experiments and are shown as % thymidine uptake when the control (HA-, Z-VAD-FMK-) uptake is regarded as 100%. The significance of differences was analyzed by Student’s *t*-test. NS, not significant. (**c**) Effects of specific inhibitors against autophagy or necrosis. KOPB26 cells (0.5 × 10^5^ per well) were cultured in triplicate in the presence or absence of ULMW-HA (2.5 mg/ml) with or without necrostatin-1 (5 *μ*M) or 3-MA (0.25 mM) for 3 days, and thymidine uptake was examined. The results are representative from three separate experiments and are shown as % thymidine uptake (mean±SE) when the control (HA-, necrostatin-1-, 3-MA-) uptake is regarded as 100%. The significance of differences was analyzed by Student’s *t*-test. NS, not significant. (**d**) Quantitative analysis of HMGB1 in culture media. KOPB26 cells (0.5 × 10^5^ per well) were cultured in the presence or absence of ULMW-HA (2.5 mg/ml) for up to 4 days, and the levels of HMGB1 in the culture supernatant were measured in triplicate by ELISA kit at days 2, 3 and 4. The culture supernatant after heating at 55 °C for 3 min was used as a positive control. The results (mean±S.E.) are representative from two separate experiments. The significance of differences was analyzed by Student’s *t*-test. NS, not significant. (**e**) Intracellular staining of HMGB1. KOPB26 cells (2.0 × 10^4^ per well) were cultured in the absence (left panels; 40 magnification) or presence (right panels; 40 and 120 magnifications) of ULMW-HA (2.5 mg/ml) for 3 days, stained with anti-HMGB1 mAb or DAP1, and observed using fluorescence microscope. Fluorescence photographs are as follows; mergence of HMGB1 and DAPI stainings (top panels), HMGB1 staining (middle panels) and DAPI staining (bottom panels), respectively

To completely rule out the possibility that ULMW-HA-induced cell death is mediated by apoptosis, we examined changes in the caspase-3 activation by flow cytometry using anti-cleaved caspase-3 antibody. The histone deacetylase inhibitor Tricostatin A was used as a positive control. As shown in Figure 5a, no cells were stained with the antibody at day 4, indicating that a machinery of activating caspases (leading to apoptosis) is not elicited in ULMW-HA-induced cell death. We next examined the effect of pan-caspase inhibitor Z-VAD-FMK (100 *μ*M) on thymidine uptake after ULMW-HA stimulation, and found no significant changes (Figure 5b), confirming that ULMW-HA-induced cell death is not elicited by induction of apoptosis. We next performed thymidine uptake assays with or without the necrosis inhibitor necrostatin-1 (5 *μ*M) and the autophagy inhibitor 3-methyladenine (3-MA; 0.25 mM). In these inhibition experiments, thymidine uptakes were pulsed at day 3, but not at day 4, after ULMW-HA stimulation, to effectively address the effect of inhibitors. As shown in Figure 5c, necrostatin-1 treatment completely restored ULMW-HA-induced suppression of thymidine uptake. In contrast, 3-MA treatment rather showed a modest enhanced effect although not statistically significant, suggesting that autophagy should be involved in survival process for avoiding necrosis. This canceling of ULMW-HA-induced suppression by necrostatin-1 treatment was also confirmed in the other cell line KOCL51 with intermediate CD44 expression (data not shown). These results strongly suggest that ULMW-HA-induced cell death is mediated by induction of necrosis.

High-mobility group box 1 protein (HMGB1) is normally a chromatin-associated protein and is sequestered at condensed chromatin in the process of apoptosis. In contrast, in cells undergoing necrotic cell death, poly(ADP)-ribose polymerase activation leads to the translocation of HMGB1 from the nucleus to the cytosol and eventually to the extracellular space if the cell loses plasma membrane integrity as a result of necrosis, where it acts as an inflammatory cytokine.^18^ We thus performed quantitative analysis of HMGB1 in the culture supernatants after ULMW-HA stimulation. The HMGB1 levels gradually increased and reached a maximal level at day 4 (99.54±4.15 ng/ml), which was much higher than that in a positive control supernatant (50.48±1.99 ng/ml) after necrosis-inducing heat (55 °C, 3 min) treatment (Figure 5d). To directly visualize the translocation of HMGB1, we performed intracellular staining of HMGB1 at day 3 after culture in the presence or absence of ULMW-HA. As shown in Figure 5e, viable leukemia cells became strongly positive for HMGB1 predominantly in the cytoplasm after ULMW-HA stimulation, suggesting that HMGB1 is actively translocated from the nucleus to the cytosol after ULMW-HA stimulation and is eventually secreted into the extracellular space. To address whether secreted HMGB1 triggered by ULMW-HA stimulation can directly kill leukemia cells, we examined changes in the viability of KOPB26 cells by dye exclusion test after 4-day culture in the presence of human recombinant HMGBI, and found that exogenously added HMGB1 itself could not kill leukemia cells even at a concentration as high as 1 *μ*g/ml.

We finally examined the ultrastructure of leukemia cells (500 cells) by transmission electron microscopy (TEM) at day 3 after ULMW-HA stimulation, and confirmed that ULMW-HA-induced cell death is mainly mediated by necrosis, because no ultrastructural findings of apoptosis and autophagy were detected in the dying cells. As representatively shown in Figure 4B, the dying cells (approximately 30% of the total cells) lost their plasma membrane integrity and showed ruptured nuclear membranes and swollen mitochondria with vacuolar cristae characteristic of necrotic cell death (Figures 4Ba and b). About 30% of the living cells had widely opened endoplasmic reticulums (ERs) (over 200 nm as the greatest diameter), and about 10% of which included autophagosomes and autolysosomes (Figures 4Bc and d) reported before as characteristic ultrastructures of ER stress-induced autophagy.^19^

### Molecular analysis of cell death after ULMW-HA stimulation

**Figure 6:**
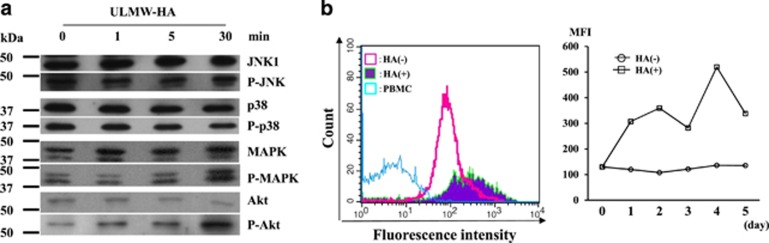
Analysis of molecular mechanisms of cell death after ULMW-HA stimulation. (**a**) Western blot analysis of changes in activation status of signal transduction molecules. KOPB26 cells (5 × 10^6^ per tube) were stimulated with ULMW-HA (10 mg/ml), and harvested at 1, 5 and 30 min. Cell lysates were separated, blotted and stained with antibodies against whole or phosphorylated form of JNK1, p38, MAPK and Akt. The bands were visualized using an enhanced chemiluminescence kit. (**b**) Flow cytometric analysis of changes in ROS production. CM-H_2_DCFDA, an intracellular ROS detector, was absorbed into KOPB26 cells, and then they (0.5 × 10^5^ per well) were cultured in the presence or absence of ULMW-HA (2.5 mg/ml) for up to 5 days. The levels of intracellular ROS were analyzed before and at days 1, 2, 3, 4 and 5 after ULMW-HA stimulation by flow cytometry. Left panel: flow cytograms of ROS production at day 3. Peripheral blood mononuclear cells (PBMC) were used as a control. The left- and right-sided open cytograms show results of PBMC and after culture without HA, respectively, whereas the filled cytogram shows the result after culture with HA. Right panel: changes in ROS production. The data (MFIs) are representative from two separate experiments

Stress-activated protein kinases, consisting of c-Jun N-terminal kinases (JNKs) and p38, are activated by their phosphorylation on specific sites after a variety of cellular stresses including necrosis-inducing heat shock and are involved in determining cell fate.^20^ p44/p42 mitogen-activated protein kinase (MAPK) and Akt are serine/threonine kinases that have an important role in multiple cellular processes including activation of proliferation and inhibition of apoptosis.^21^ We thus examined on western blots whether JNK1, p38, MAPK and Akt were activated after ULMW-HA stimulation (10 mg/ml). As shown in Figure 6a, JNK1 and p38 were not phosphorylated. In contrast, MAPK and Akt were markedly phosphorylated at 30 min after stimulation, suggesting that the activation signal is temporarily transmitted via CD44–ULMW–HA interaction.

As enhanced reactive oxygen species (ROS) production is frequently involved in induction of cell death in the process of necrosis, as well as apoptosis,^22^ we examined changes in intracellular production of ROS after ULMW-HA stimulation (2.5 mg/ml) by flow cytometry, and found that the ROS level gradually increased and reached a maximal level at day 4, approximately fivefold higher when compared with that before the stimulation (Figure 6b), suggesting that upregulated ROS production is likely associated with ULMW-HA-induced necrotic cell death.

**Figure 7:**
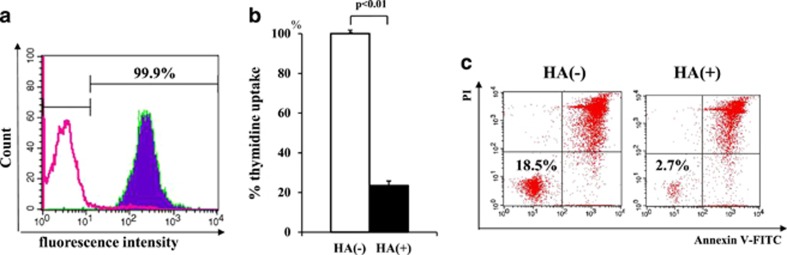
Analysis of primary *MLL*+ALL cells from case 4**.** (**a**) Surface expression of CD44. Leukemia cells were stained by FITC-conjugated anti-CD44 antibody (filled cytogram) or murine IgG1 (control, open cytogram), and analyzed by flow cytometry. (**b**) Changes in thymidine uptake after ULMW-HA stimulation. Leukemia cells (1 × 10^5^ per well) were cultured in the presence or absence of ULMW-HA (2.5 mg/ml) for 4 days, and thymidine uptake were measured. (**c**) Changes in viability on flow cymometer after ULMW-HA stimulation. Leukemia cells (1 × 10^5^ per well) were cultured in the presence or absence of ULMW-HA (2.5 mg/ml) for 4 days, and stained doubly with annexin V-FITC and PI. Double-negative (lower left) cell population was regarded as viable

We finally examined whether or not primary B-precursor ALL cells also show similar results after ULMW-HA stimulation. For this purpose, primary leukemia cells (>90% blasts) from six cases of B-precursor ALL, which have been stored in liquid nitrogen were thawed and cultured for 4 days in the presence or absence of ULMW-HA (2.5 mg/ml), and their thymidine uptakes and cell death analysis on flow cytometer were performed. As representatively shown in Figure 7, primary *MLL*+ALL cells from case 4 showed the high CD44 expression (A) and a marked inhibition of thymidine uptake (B) and a decrease in viability by flow cytometric analysis (C) in the presence of ULMW-HA. Results were summarized in Table 1. Primary B-precursor ALL cells from five cases with high CD44 expression, but not those from one case with low CD44 expression, were significantly inhibited in their viabilities. These results strongly suggest that ULMW-HA-triggered cell death is elicited not only in established cell lines but also in primary leukemia cells when they show B-precursor lineage and high CD44 expression.

## Discussion

The content of HA in many normal biological fluids has been determined, and its concentration in synovial fluid of the human knee joint is reported to be very high (2–3 mg/ml) to provide viscoelasticity and lubrication necessary for protection of cartilage surface.^23^ There are a number of studies showing increased HA content, but reduced average molecular mass with a broader range of sizes in tissues or fluids when inflammatory or tissue-remodeling processes occur.^23^ In fact, the HA concentration in human serum is reported to be elevated in inflammatory diseases such as rheumatoid arthritis (maximally up to 200 *μ*g/ml),^24^ although its concentration in healthy individuals is very low (usually <40 ng/ml). Of importance, 4.3 kDa LMW HA (defined as ULMW-HA in this study) is reported to induce proinflammatory cytokine IL6 and several chemokines in human dermal fibroblasts in a CD44-dependent manner,^25^ suggesting that highly degraded HA with a very low-molecular size produced at the site of inflammation is thought to potentiate inflammatory processes. Although there have been no reports until now describing concentrations of ULMW-HA in human serum, tissues and fluids, its concentration at the specific inflammatory sites is postulated to be very high. In this regard, the concentration of ULMW-HA (2.5 mg/ml) used in this study should be within ranges in local and particularly pathological circumstances. Moreover, it should be noted that a temporal exposure to a high concentration of ULMW-HA is enough for leukemia cells to undergo cell death.

We first showed in this study that ULMW-HA markedly induced necrotic cell death of B-precursor ALL cells with high surface CD44 expression, in the process of which a large amount of HMGB1 was translocated from the nucleus to the cytoplasm and then released to the extracellular space. The release of HMGB1 has been regarded as a necrosis marker,^26, 27^ but it was recently shown to be released by necroptosis, a newly recognized, regulated form of necrosis, as one of proinflammatory damage-associated molecular patterns (DAMPs), which promote inflammatory responses through interactions with Toll-like receptors and other innate receptors.^28^

The ULMW-HA-induced cell death was completely blocked by necrosis inhibitor necrostatin-1, leading to the conclusion that ULMW-HA kills B-precursor ALL cells by induction of necrosis. Autophagy is a major intracellular degradative process that delivers cytoplasmic materials to the lysosome for degradation, and mutations of autophagy-related genes have been recently identified in various human diseases,^29^ suggesting its pivotal role for avoiding cell damage, but not a form of cell death. Findings obtained from TEM also demonstrated that the ULMW-HA-induced cell death was primarily mediated by necrosis, and induction of autophagy observed in a small population may be a survival mechanism from the cell death-inducing insult by ULMW-HA.

We also showed that the ROS level gradually increased after ULMW-HA stimulation, suggesting that upregulated ROS production is likely associated with ULMW-HA-induced necrotic cell death. Recently, overproduction of ROS has attracted much attention as a factor that induces tumor cell death, because it has been reported that some cancer stem cells maintain their characteristics by inhibiting production of ROS to avoid cell death.^30^ Although necrostatin-1 has no intrinsic antioxidant activity, it is reported to block tumor necrosis factor-induced activation of NADPH oxidase and nitric oxide-mediated necrosis by inhibiting both Nox1 and mitochondrial-derived oxygen-free radicals.^31^ It is also reported that necrostatin-1 blocks programmed necrosis by inhibiting receptor-interacting protein kinases (RIPK)-1 and 3 and is significantly neuroprotective in adult and neonatal rodent models of ischemic brain injury.^32, 33^ Thus, it is conceivable that necrostatin-1 inhibits ULMW-HA-induced necrosis by counteracting intracellular overproduction of ROS and suppressing RIPK-1 and 3 activity.

We examined on western blots if several molecules involving signal transduction pathways were activated after ULMW-HA stimulation, and found that p44/p42 MAPK and Akt were phosphorylated. As activation of both p44/p42 MAPK and Akt inhibits not only apoptosis, but also autophagy via activation of the mammalian target of rapamycin,^34^ their activation should be involved in determining the mode of cell death, thus leading to necrosis, but not to apoptosis, after ULMW-HA stimulation.

It has been clinically observed that several neoplasms including leukemia mysteriously show spontaneous regression after severe feverish infection and inflammation.^35, 36^ As ULMW-HA is abundantly secreted from mesenchymal cells at the site of infection and inflammation,^37^ it may directly act as one of cell death-inducing factors for tumors expressing CD44 at high levels. In addition, although HMGB1, abundantly released from CD44-high leukemia cells after ULMW-HA stimulation can not directly kill leukemia cells, it should act as one of proinflammatory DAMPs and stimulate to secrete ULMW-HA from mesenchymal cells, which in turn could induce cell death of CD44-high leukemia cells with a release of HMGB1, thus leading to the formation of cell death-inducing positive feedback loop for leukemia cells at the site of infection and inflammation. This may provide a new insight for understanding mechanism(s) of spontaneous regression of tumors after febrile and inflammatory episodes.

Although clinical application of ULMW-HA itself for the treatment of surface CD44-high B-precursor ALL cases would not be realistic because of its required high concentration in serum and predictable adverse events, CD44-targeted approaches will possibly become a useful treatment strategy not only for B-precursor ALL, but also for surface CD44-high hematological and non-hematological tumors if specific small compound(s) or anti-CD44 monoclonal antibody capable of mimicking the action of ULMW-HA could be successfully explored. Such agents should be very effective by predominantly eradicating CD44-positive leukemia- or cancer-initiating cells.

## Materials and Methods

### Leukemia cells

Eight *MLL*+ALL cell lines (three *MLL-AF4*: KOCL45, KOCL58, KOCL69; two *MLL-AF9*: KOPB26, YACL95; three *MLL-ENL*: KOPN1, KOCL33, KOCL51), 10 B-precursor ALL cell lines (four Philadelphia chromosome-positive: KOPN30bi, KOPN55bi, KOPN57bi, KOPN66bi; four *TCF3-PBX1* positive: KOPN34, KOPN36, KOPN54, YAMN92; two *ETV6-RUNX1*: KOPN41, REH) and 5 T-ALL cell lines (JURKAT MOLT4, KOPTK1, KOPT5 and KOPT11), which have been described previously,^4^ were used for examining surface CD44 expression and thymidine uptake. The EBV-transformed B lymphoid cell line YAMB9 was also used for comparison. The cell line KOPB26 (fusion transcript *MLL-AF9*) with very high surface expression of CD44 was extensively used throughout the study. Six samples of primary ALL cells (>90% blasts), which have been stored in liquid nitrogen were thawed and used for some experiments.

### Establishment of the cell line expressing CD44 at very low levels by targeted genome editing

KOPB26 cell line expressing CD44 at very low levels was established using the clustered regularly interspaced short palindromic repeat (CRISPR)/ CRISPR-associated protein-9 nuclease (Cas9) system^16^ according to the manual of GeneArt CRISPR Nuclease Vector Kit (Life Technologies, Carlsbad, CA, USA). Guide RNAs for human *CD44 exon 2* (sense: 5′-CTACAGCATCTCTCGGACGGgtttt-3′, and antisense: 5′-CCGTCCGAGAGATGCTGTAGcggtg-3′) were inserted into CRISPR nuclease CD4 vector, and transfected into the parent cell line by Neon Transfection System (Life Technologies). The CD4-positive cells were collected using CD4-microbeads (Miltenyi Biotec, Auburn, CA, USA) 3 days after transfection, and then CD44-negative cells were selected by anti-CD44 murine monoclonal antibody (mAb; Immunotech, Vaudreuil-Dorjon, Quebec, Canada) and rabbit anti-mouse antibody-conjugated immunomagnetic beads. Extracted genomic DNA from this cell line was amplified by PCR using primers 5′-TAACCTGCCGCTTTGCAGGTGTATT-3′ (sense) and 5′-GCCATTGTGGGCAAGGTGCTATTGA-3′ (antisense) for human *CD44 exon 2*, and the PCR products were inserted into the pGEM-T Easy vector (Promega, Madison, WI, USA) and introduced into bacteria. The inserted fragments derived from the individual PCR amplicons in each clone were sequenced by Sanger method.

### Reagents and antibodies

HMW-HA (10^3^–10^4^ kD) and ULMW-HA (4–8 kD) were purchased from R&D Systems (Minneapolis, MN, USA). Human recombinant HMGB1 was purchased from Prospec (East Brunswick, NJ, USA). The ROS detector CM-H_2_DCFDA (5-chloromethyl-2′7′-dichlorohydro-fluorescein diacetate) was purchased from Life Technologies. Z-Val-Ala-Asp(OMe)-FMK (Z-VAD-FMK), methylthiohydantoin-DL-tryptophan (necrostatin-1) and 3-MA were purchased from Enzyme Systems Products (Livemore, CA, USA), Enzo Life Sciences (Farmingdale, NY, USA) and Calbiochem (La Jolla, CA, USA), respectively. Murine FITC-conjugated anti-CD44 monoclonal antibody (mAb) (J.173, IgG1) was purchased from Beckman Coulter (Brea, CA, USA). PE-conjugated rabbit anti-cleaved caspase-3 antibody and anti-HMGB1 mAb were purchased from BD Biosciences (San Jose, CA, USA). Other mAbs against p44/p42 MAPK, phosphorylated MAPK (Thr^202^/Tyr^204^), Akt, phosphorylated Akt (Ser^473^), p38, phosphorylated p38 (Thr^180^/Tyr^182^), JNK1 and phosphorylated JNK1 (Thy^183^/Tyr^185^) were purchased from Cell Signaling Technology (Beverly, MA, USA). Rabbit anti-RHAMM (receptor for HA-mediated motility, CD168) antibody was purchased from Lifespan Biosciences (Seattle, WA, USA). Rat mAb specifically recognizing human CD44v9 (clone RV3) was from Cosmo Bio (Tokyo, Japan).

### Thymidine uptake analysis

Leukemia cells (2.5–5.0 × 10^4^ per well) were cultured in RPMI-1640 medium supplemented with 7.5% fetal calf serum (FCS) in a 96-well flat-bottomed culture plate in triplicate in the presence or absence of various concentrations of HA at 37 °C for the indicated periods of time, and 5 h-[^3^H]-thymidine uptakes were measured. The % inhibition of thymidine uptake was calculated as follows; {1–[(cpm of treated cells)/(cpm of untreated cells)]} × 100. The % thymidine uptake was defined as [(cpm of treated cells)/(cpm of untreated cells)] × 100. In some experiments, cells were cultured with or without one of several reagents.

### Dye exclusion test

Leukemia cells (5 × 10^4^ per well) were cultured in RPMI-1640 medium supplemented with 7.5% FCS in a 96-well flat-bottomed culture plate in the presence or absence of ULMW-HA (2.5 mg/ml) at 37 °C for the indicated periods of time. The numbers of living and dead cells were counted by the dye exclusion test and their viability (%) was calculated.

### Cell cycle analysis

Leukemia cells (5 × 10^4^ per well) were cultured in RPMI-1640 medium supplemented with 7.5% FCS in the presence or absence of ULMW-HA (2.5 mg/ml) for 2–4 days. These cells were washed and suspended in 0.1% Triton X-PBS, and then treated with RNase at 37 °C for 15 min. The cells treated with PI (10 *μ*g/ml) were analyzed using a flow cytometer (FACSCalibur, BD Biosciences).

### Flow cytometric analysis

Leukemia cells were stained directly or indirectly with the control normal IgG or the specific antibody such as anti-CD44 and anti-CD44v9. In some experiments, leukemia cells (5 × 10^4^ per well) were cultured in RPMI-1640 medium supplemented with 7.5% FCS in the presence or absence of ULMW-HA (2.5 mg/ml) for 2–4 days. Cells were then harvested and stained doubly with FITC-conjugated annexin V and PI (1 *μ*g) for 15 min in the dark. Ten thousand events were analyzed using a flow cytometer.

### Measurement of high-mobility protein group B1 (HMGB1) in culture media

Leukemia cells (2 × 10^4^ per well) were cultured in RPMI-1640 medium supplemented with 7.5% FCS in the presence or absence of ULMW-HA (2.5 mg/ml) up to 4 days, and the level of HMGB1 in the culture supernatant was measured with an ELISA kit from Shinotest (Sagamihara, Kanagawa, Japan) at days 2, 3 and 4. The culture supernatant from cells after heating at 55 °C for 3 min was used as a positive control.

### Intracellular staining of HMGB1

Leukemia cells (2 × 10^4^ per well) were cultured in RPMI-1640 medium supplemented with 7.5% FCS in the presence or absence of ULMW-HA (2.5 mg/ml) for 3 days. Cells were then fixed and permeabilized with PBS containing 4% paraformaldehyde and saponin (Cytofix/Cytoperm solution, BD Biosciences), and stained with anti-HMGB1 mAb or DAP1 (4′,6-diamidino-2-phenylindole; Dojindo, Kumamoto, Japan). Cells were sealed in the mounting agent (Fluoromount/Plus, Diagnostic BioSystems, Pleasanton, CA, USA) and observed using the phase-contrast fluorescence microscope (BZ-9000, Keyence, Osaka, Japan).

### TEM examination of cell death

Leukemia cells (5 × 10^4^ per well) were cultured in RPMI-1640 medium supplemented with 7.5% FCS in the presence or absence of ULMW-HA (2.5 mg/ml) for 3 days. The cells were fixed with 2.5% glutaraldehyde and 1% osmium tetroxide, dehydrated in a graded series of ethanol, and finally embedded in Epon 812 epoxy resin. The ultrathin sections at 70–80 nm were mounted on copper grids and doubly stained with uranyl acetate and lead citrate. They were finally observed at an accelerating voltage of 80 kV in TEM (H-7500, Hitachi, Tokyo, Japan). Five hundred cells selected at random were analyzed.

### Western blot analysis

Lysates of leukemia cells were separated on a SDS-polyacrylamide gel and transferred to nitrocellulose membranes as reported previously,^4^ which were incubated with various primary antibodies at 4 °C overnight, and then with horseradish peroxidase-conjugated secondary antibody at room temperature for 1 h. The bands were visualized using an enhanced chemiluminescence kit (Amersham, Buckinghamshire, UK).

### ROS production

CM-H_2_DCFDA (5 *μ*M), an intracellular ROS detector, was absorbed into leukemia cells at 37 °C for 30 min, and then they (5 × 10^4^ per well) were cultured in RPMI-1640 supplemented with 7.5% FCS in the presence or absence of ULMW-HA (2.5 mg/ml) for 5 days. Changes in the production of intracellular ROS were analyzed by flow cytometry.

### Analysis of CD44 splice variants

Competitive reverse transcription-PCR analysis of CD44 splice variant RNAs in human ALL cell lines was performed with the use of Takara Premix Taq DNA Polymerase (Takara Bio, Shiga, Japan), and the PCR products were fractionated by agarose gel electrophoresis. The following human primer sets (forward and reverse, respectively) were used: CD44, 5′-TCCCAGACGAAGACAGTCCCTGGAT-3′ and 5′-CACTGGGGTGGAATGTGTCTTGGTC-3′ and *β*-actin, 5′-AGGCACCAGGGCGTGAT-3′ and 5′- GCCCACATAGGAATCCTTCTGA-3′, as reported previously.^17^

### Statistics

Unpaired *t*-test was used for the comparison of the differences in [^3^H]-thymidine uptake and in the level of HMGB1. A *P*-value of <0.05 was considered significant.

## Figures and Tables

**Table 1:** Characteristics of primary leukemia cells and induction of cell death after ULMW-HA stimulation

**Case no.**	**Age (year)/sex**	**WBC (× 10**^**3**^**/*****μ*****l)**	**Sample**	**Fusion gene**	**CD44 expression**		**% Decrease in viability after ULMW-HA stimulation**
					**%**	**MFI (fold)**	
1	0.7/F	824	PB	*MLL-AF4*	98.9	259.5	50.2
2	0.3/F	183	PB	*MLL-ENL*	98.8	395.1	41.6
3	0.5/M	700	PB	*MLL-AF4*	99.9	452.1	47.9
4	2.3/M	327	PB	*MLL-AF9*	99.9	214.0	85.1
5	7.2/F	11.4	BM	*ETV6-RUNX1*	99.9	265.5	66.2
6	5.7/M	15.4	BM	*TCF3-PBX1*	95.7	21.1	14.2

Abbreviations: BM, bone marrow; F, female; M, male; MFI, mean fluorescence intensity; PB, peripheral blood. MFI (fold); MFI (stained by FITC-conjugated anti-CD44 antibody)/MFI (stained by murine IgG1). % Decrease in viability after ULMW-HA stimulation; {1– [(lower left population% with HA)/(lower left population% without HA)]} × 100
