# Supplementary material for: Inflammatory mediator ultra-low-molecular-weight hyaluronan triggers necrosis of B-precursor leukemia cells with high surface CD44 expression
Source: Cell Death Dis. 2017 Jun 1;8(6):e2857–. doi: 10.1038/cddis.2017.249 (PMC5520907; doi:10.1038/cddis.2017.249)
Supplement: Supplementary Figure 1 [file cddis2017249x1.pdf]

Supplementary figure 1

Genomic DNA (Exon 2) of CD44

Target PAM

5′ ATGGTCGCTACAGCATCTCTCGGACGGAGGCCGCTGA 3′

(a) Partial sequences of clones

WT: 5′ ATGGTCGCTACAGCATCTCTCGGACGGAGGCCGCTGA 3′
Clone 1: 5′ ATGGTCGCTACAGCATCTCTCGGACGGAGGCCGCTGA 3′
Clone 2: 5′ ATGGTCGCTACAGCATCTCTCGGA**CCA**CGGAGGCCGCTGA 3′
Clone 3: 5′ ATGGTCGCTACAG---------GACG-------CTGA 3′
Clone 4: 5′ ATGGTCGCTACAGCATCTCTCGGA**AAC**CGGAGGCCGCTGA 3′
Clone 5: 5′ ATGGTCGCTACAGCATCTCTCGGA**GGG**CGGAGGCCGCTGA 3′
Clone 6: 5′ ATGGTCGCTACAGCATCTCTCGGA**TTC**CGGAGGCCGCTGA 3′
Clone 7: 5′ ATGGTCGCTACAGCATCTCTCGGA**CGGC**CGGAGGCCGCTGA 3′

(b) Predicted amino acid sequences

WT: 28-TCRFAGVFHVEKNGRYSISRTEAADLCKAFNSTLPTM
Clone 1: 28-TCRFAGVFHVEKNGRYSISRTEAADLCKAFNSTLPTM
Clone 2: 28-TCRFAGVFHVEKNGRYSISRT**T**EAADLCKAFNSTLPTM
Clone 3: 28-TCRFAGVFHVEKNGRY**RTLTSARLSIAPCPQWPRWRKL***
Clone 4: 28-TCRFAGVFHVEKNGRYSISR**KP**EAADLCKAFNSTLPTM
Clone 5: 28-TCRFAGVFHVEKNGRYSISR**RA**EAADLCKAFNSTLPTM
Clone 6: 28-TCRFAGVFHVEKNGRYSISR**IP**EAADLCKAFNSTLPTM
Clone 7: 28-TCRFAGVFHVEKNGRYSISRT**AGGR***
